# Intelligent Simultaneous Quantitative Online Analysis of Environmental Trace Heavy Metals with Total-Reflection X-Ray Fluorescence

**DOI:** 10.3390/s150510650

**Published:** 2015-05-06

**Authors:** Junjie Ma, Yeyao Wang, Qi Yang, Yubing Liu, Ping Shi

**Affiliations:** 1 School of Water Resources & Environment, China University of Geosciences (Beijing), Beijing 100083, China; E-Mails: majunjie85@gmail.com (J.M.); yeyaowang@163.com (Y.W.); 2 China National Environmental Monitoring Centre, Beijing 100012, China; 3 Yiwen Environmental Science Technology Co., Ltd, Guangzhou 510730, China; E-Mails: liuyb@yiwenkeji.com (Y.L.); shiping@yiwenkeji.com (P.S.)

**Keywords:** total-reflection X-ray fluorescence, on-line analysis, quantitative determination, particle swarm optimization, radial basis function network, simulated annealing

## Abstract

Total-reflection X-ray fluorescence (TXRF) has achieved remarkable success with the advantages of simultaneous multi-element analysis capability, decreased background noise, no matrix effects, wide dynamic range, ease of operation, and potential of trace analysis. Simultaneous quantitative online analysis of trace heavy metals is urgently required by dynamic environmental monitoring and management, and TXRF has potential in this application domain. However, it calls for an online analysis scheme based on TXRF as well as a robust and rapid quantification method, which have not been well explored yet. Besides, spectral overlapping and background effects may lead to loss of accuracy or even faulty results during practical quantitative TXRF analysis. This paper proposes an intelligent, multi-element quantification method according to the established online TXRF analysis platform. In the intelligent quantification method, collected characteristic curves of all existing elements and a pre-estimated background curve in the whole spectrum scope are used to approximate the measured spectrum. A novel hybrid algorithm, PSO-RBFN-SA, is designed to solve the curve-fitting problem, with offline global optimization and fast online computing. Experimental results verify that simultaneous quantification of trace heavy metals, including Cr, Mn, Fe, Co, Ni, Cu and Zn, is realized on the online TXRF analysis platform, and both high measurement precision and computational efficiency are obtained.

## 1. Introduction

Total-reflection X-ray fluorescence (TXRF) analysis has made great progress in recent years, applied in diverse fields such as environmental, medical, industrial, biological, and food analysis [1,2,3]. TXRF analysis is an energy-dispersive X-ray fluorescence (EDXRF) technology adopting unique excitation geometry. It has advantages of simultaneous multi-element analysis capability, decreased background noise, no matrix effects, wide dynamic range, ease of operation, and the potential of trace analysis. Simultaneous quantitative online analysis of trace heavy metals in the environment, especially in natural water, is urgently required, since heavy metals from natural (e.g., minerals and rocks) or anthropogenic (e.g., industrial, agricultural, and urban activities) origin have possible harmful effects to consumers, and dynamic monitoring of heavy metals is necessary for health and environmental authorities. Considering simultaneous multi-element analysis capability of TXRF, we can extend its application to online monitoring, which has not been explored yet. A scheme consisting of a TXRF-based online analysis system should be investigated. There are still problems in practical quantitative TXRF analysis, especially in quantitative online TXRF analysis. For measured spectra, overlapping often occurs between characteristic peaks of multi-elements from different sources, owing to the finite energy resolution of detectors. Also, although spectral background is decreased because of total reflection, it may drift from measurement to measurement due to subtle changes of testing conditions. These problems can create real difficulties for quantitative multi-element determination, especially for elements of low concentration. Therefore, a corresponding quantification method should be developed, considering the overlapping and background effects, and also considering the demands of online monitoring applications, including high robustness and timeliness.

The fundamental principle of quantitative TXRF analysis is that a linear relationship exists between the concentration of certain element and its X-ray intensity recorded. In traditional quantification approaches, one single element as an internal standard is added to the sample at a known concentration. Then the unknown concentration of other elements can be determined using detection sensitivities of the analyte and the internal standard, and also the net intensity of the analyte. The way to extract the net intensities of characteristic peaks from each spectrum follows the common approaches in EDXRF. Conventionally, an independent background correction process, such as stripping-peak, is applied in advance in order to find the spectral background. Then, the principal peaks are used in calculation of net intensities directly. In a few cases, the overlapping among these principal peaks is considered, and a few nearby peaks from related elements are studied at the same time to fit the local spectrum window. However, these traditional quantification approaches consider only local spectral information of a few elements for calculation, and carry risks of over-fitting and under-fitting of spectral background. Overlapping of unknown elements as well as background drifting may lead to loss of accuracy or even faulty results. More importantly, since these traditional approaches essentially rely on experience, they are difficult to generalize or use in online TXRF analysis.

Focused on the mentioned problems, we firstly established an online TXRF analysis platform, on which sample preparation, TXRF measurement and carrier cleaning are performed automatically and periodically, according to the programmed procedure. Based on the online TXRF analysis platform, we present an intelligent quantification method. In this method, a new spectral decomposition approach of measured spectra is utilized, taking into account the characteristic curves of possible elements in the whole spectrum scope, as well as the pre-estimated spectral background. Then, the quantification problem becomes an optimization problem, searching for the best combination of characteristic curves and one background curve to fit the measured spectrum. Considering the benefits of particle swarm optimization (PSO) in solving combinatorial optimization problems [4,5], the benefits of radial basis function network (RBFN) in approximating non-linear function and performing fast computation [6], and the benefits of simulated annealing (SA) in finding the local optimal result [7], a novel hybrid algorithm, PSO-RBFN-SA, is well designed for online TXRF analysis to ensure measurement accuracy and save computing time. The framework of this method is scalable and new elements can be added easily if needed. It has good potential to be extended to other kinds of samples and applications. Simultaneous quantitative on-line TXRF analysis of trace heavy metals is performed, and experimental results demonstrate that the proposed intelligent quantification method can achieve high measurement precision and computational efficiency.

The rest of this paper is organized as follows. Section 2 presents the related work of this research. Section 3 introduces the establishment of our online TXRF analysis platform. In Section 4, the intelligent quantification method for online TXRF analysis is explained in detail. The influence factors in quantitative multi-element determination are described, and the spectral decomposition framework is introduced. Then, the objective function is formulated for the combinatorial optimization problem, and the PSO-RBFN-SA algorithm is discussed. The experimental results are presented in Section 5, where we run the online TXRF analysis and evaluate the intelligent quantification method. We conclude the paper in Section 6.

## 2. Related Work

Studies of total-reflection X-ray fluorescence (TXRF) have been performed, and remarkable progress has been made in recent years. As a promising analytical technology, great efforts have been made on basic components, experimental conditions, performance evaluation, sample pretreatment, environmental samples, novel working modes, *etc.*, as shown in Table 1.

**Table 1:** Recent researches in the field of TXRF.

Research Area	Main Work	References
Basic components	Compact system construction	[8,9]
	Parallel primary beam	[10]
	Polycapillary semi-lens	[11]
Experimental conditions	Glancing angle optimization	[12,13]
	Flowing nitrogen gas during detection	[14]
Performance evaluation	Evaluation of accuracy, limits, *etc.*	[15,16,17,18]
Sample pretreatment	Direct treatment, mineralization, extraction, *etc.*	[19]
	Ideal sample shape	[20]
	Pre-concentration	[21,22]
	Avoiding Hg volatilization	[23]
	Ultrasound-assisted extraction	[24]
	Vermicompost as adsorbent substrate	[25]
Environmental applications	Analysis of environmental samples, *etc.*	[26,27,28,29,30,31,32,33,34,35,36,37,38,39,40,41,42,43,44,45,46,47,48]
Novel working modes	μ-TXRF	[49]
	Sweeping-TXRF	[50]
	Related applications	[51,52,53]

First of all, there are many research results on basic components of TXRF. In [8,9], the construction of a simple and precise TXRF spectrometer is discussed. In particular, use of parallel primary beam and polycapillary semi-lens is studied in [10,11], respectively. Furthermore, Bernasconi *et al.* investigated experimental condition configuration with computer-assisted control of the reflector position [12], Kunimura *et al.* optimized the glancing angle for simultaneous trace elemental detection [13], while flowing nitrogen gas is introduced to enhance the sensitivity of TXRF in [14]. Moreover, the performance of TXRF is evaluated for ultra-trace element analysis in [15,16], while theoretical calculations of detection limits for different TXRF methods are presented in [17]. The results of the determination of Rb, Sr, Cs, Ba, and Pb in small sample amounts are presented in [18].

Secondly, many researchers pay attention to sample pretreatment strategies. In [19], the means of minimal treatment, preparation of slurries, mineralization, direct treatment on the sample carrier, extraction, speciation and fractionation are given in detail. Horntrich *et al.* studied the production of the ideal TXRF sample shape [20]. Pre-concentration with Ammonium Pyrrolidinedithiocarbamate is discussed in [21], while the specific pre-concentration method with alumina for determination of As is proposed in [22]. Besides, a chemical strategy to avoid Hg volatilization is used in [23]. Also, ultrasound-assisted extraction (UAE) was employed for acceleration of metal extraction from soil samples in [24], and vermicompost is adopted as adsorbent substrate for removing Pb, Ni, V and Cr from wastewaters in [25].

Thirdly, analysis of environmental samples with TXRF is well studied [26]. A summary of water, air dust, organ tissue and plant material analysis is shown in [27]. Borgese *et al.* ran the round-robin test of water samples [28], while Pashkova *et al.* discussed the effect of factors such as the surface density of dried water residue on the sample carrier, the dilution ratio of high-mineralized samples with ultrapure water, the salt content, and the internal standard concentration, in water sample analysis [29]. Environmental and food samples have been especially widely tested, including rainwater [30], tree-rings [31], fine roots of Scots pine [32], industrial wastewater [33], water samples during deionized water production [34], metallurgical slag [35], heavy metal contaminated soils and sediments [36], Langmuir monolayers on water surface [37], Madeira wine [38], and honey from different regions [39]. Also, TXRF is applied to analysis of surface water or ground water samples from specific regions [40,41,42,43,44,45,46,47,48].

In addition, novel working modes of TXRF for different applications have been developed. The concepts of micro total-reflection X-ray fluorescence (μ-TXRF) [49] and sweeping total-reflection X-ray fluorescence (Sweeping-TXRF) [50] have been proposed. Related applications, such as wafer analysis [51], VLSI micro contamination detection [52], and the introduction of novel materials in clean-room production [53], are extended.

However, online analysis based on TXRF is not well explored, and any related scheme of online TXRF analysis has not been proposed yet. Online TXRF analysis will be useful for dynamic monitoring and management of environmental trace heavy metals, and also will have potential to improve work efficiency of different applications. Besides, unpolished approaches of quantitative multi-element determination are adopted in most research efforts and applications. Since treatment of spectral overlapping and background essentially relies on experience, these traditional approaches are difficult generalize or use in online TXRF analysis.

The following three aspects constitute the main differences between our work and previous research: (1) a feasible scheme of online TXRF analysis is firstly proposed, and quantitative online analysis methods can be evaluated on the established online TXRF analysis platform; (2) a novel spectral decomposition framework is clearly proposed, utilizing characteristic curves of all possible elements and a pre-estimated background curve in the whole spectrum scope to approximate the measured spectrum, which is scalable and can be easily generalized; and (3) a hybrid algorithm, PSO-RBFN-SA, is well designed for online TXRF analysis in order to ensure measurement accuracy and save computing time, with offline global optimization and fast online computing.

## 3. Online TXRF Analysis Platform

**Figure 1:**
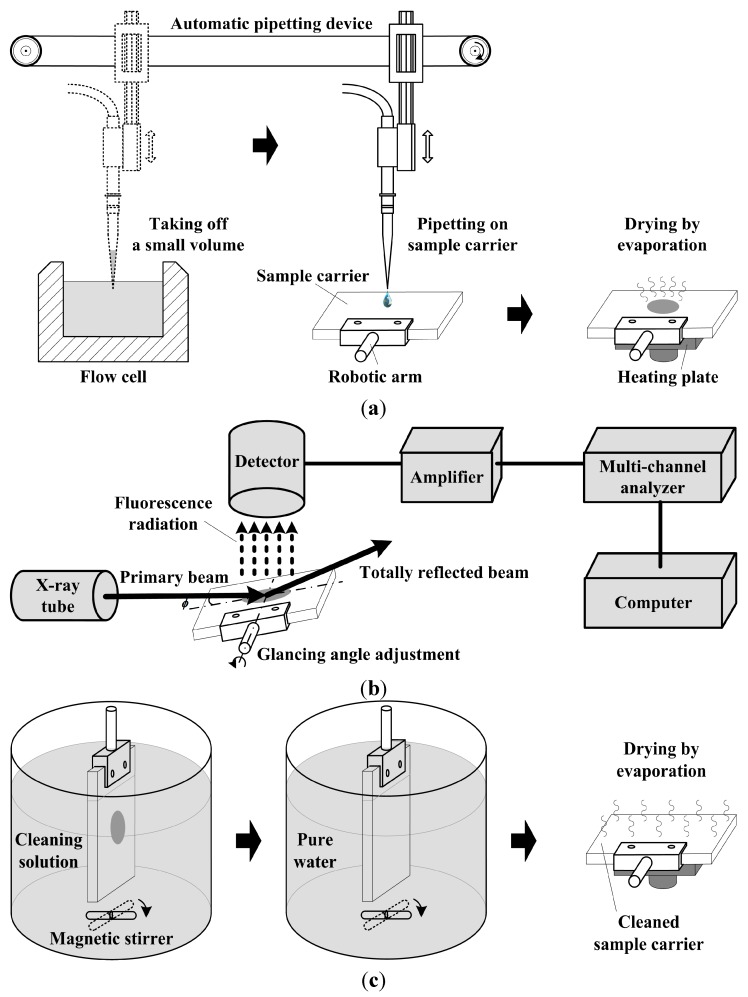
Operation procedure of the online TXRF analysis platform. (**a**) Sample preparation; (**b**) TXRF measurement; (**c**) Carrier cleaning.

One of the drawbacks of conventional EDXRF that has hampered analysis of trace element levels in small samples has been the high background due to scatter from a sample. An analytical note was originally published in *Review of Scientific Instruments* by Yoneda and Horiuchi in 1971, in which the benefits of the total reflection of X-rays at an optically flat surface for spectrochemical analysis was reported. The basic principle was that if a low, divergent, almost parallel X-ray beam impinges on the flat, smooth surface of a reflector at an angle smaller than the critical angle, total reflection occurs. Under such conditions, most of the primary beam radiation is reflected and thus the spectral background is clearly reduced. Meanwhile, the fluorescence signal is also enlarged as the reflected beam contributes to excitation, leading to a doubling in the intensity of the fluorescence signal [54,55]. Considering the simultaneous multi-element analysis capability of TXRF, we design and construct an online analysis system based on TXRF, which we call an “online TXRF analysis platform”. Natural water containing multi-elements, as the main test subject, are discussed in this paper. Figure 1 presents the operation procedure of the online TXRF analysis platform, which gives the means of sample preparation, TXRF measurement and carrier cleaning. All these steps are expected to be accomplished in each single measurement period.

Figure 1a shows the online sample preparation steps for TXRF measurement. With an automatic pipetting device, a small volume of water sample, usually 5~20 μL, is taken from the flow cell, and then pipetted onto the center of a cleaned quartz sample carrier. The flow cell is filled with test subject, pumped natural water, of which the element concentrations are changing dynamically. The quartz sample carrier is gripped by a robotic arm so that it is moveable. The droplet on the quartz sample carrier is quickly dried by evaporation via placing the sample carrier on a heating plate. For low concentration samples, the pipetting and drying process is repeated. In our case, a 10 μL water sample is taken three times, *i.e.*, totally 30 μL of water sample is used for sample preparation. The residue on the sample carrier, presented as a specimen, can then be tested by TXRF.

In Figure 1b, a classic system of TXRF analysis is employed to test the specimen. The system is mainly comprised of an X-ray tube, a detector, an amplifier, a multi-channel analyzer and a computer. The attachments of the system are designed to be freely configurable. Different X-ray tubes (e.g., Cu, Mo, W and Ag tubes) and different filters can easily be equipped. In this paper, an Ag-anode X-ray tube is used. The system utilizes a Peltier cooled solid-state detector for energy-dispersive X-ray fluorescence measurements. With the robotic arm, the sample carrier is placed at the test position and the glancing angle is adjustable. The sample carrier can be tilted around a horizontal axis in a stepwise manner. The glancing angle controller works at a resolution of 0.03°. The material of the sample carrier is quartz, and the critical glancing angle can be calculated as:

(1) $$\phi_{crit}≐\frac{1.65}{E}\sqrt{\frac{Z}{A}\rho }$$

where E represents photon energy, Z is the atomic number of the reflector, A is the atomic mass, and ρ is the density. With a certain glancing angle, the primary beam radiation is totally reflected. The fluorescence signal is conditioned and acquired. The measured spectrum of fluorescence signal should be processed to realize quantitative multi-element determination.

Figure 1c shows the on-line carrier cleaning steps after TXRF measurement. Contaminations on the sample carrier will result in errors in both qualitative and quantitative element determination. As the sample carrier plays an important role in the achievement of optimal analytical results, a proper cleaning of its surface has to be ensured to avoid memory effects, particularly if trace elements have to be determined. The used sample carrier is dipped into the cleaning solution under 90 °C with string for 15 min. The cleaning solution is the mixture of 10% sulfuric acid solution and 6 mmol/L potassium permanganate solution. Then, it is washed in pure water for one minute. Finally, the drying step is performed on the heating plate and the cleaned sample carrier is ready for the next round of sample preparation.

**Figure 2:**
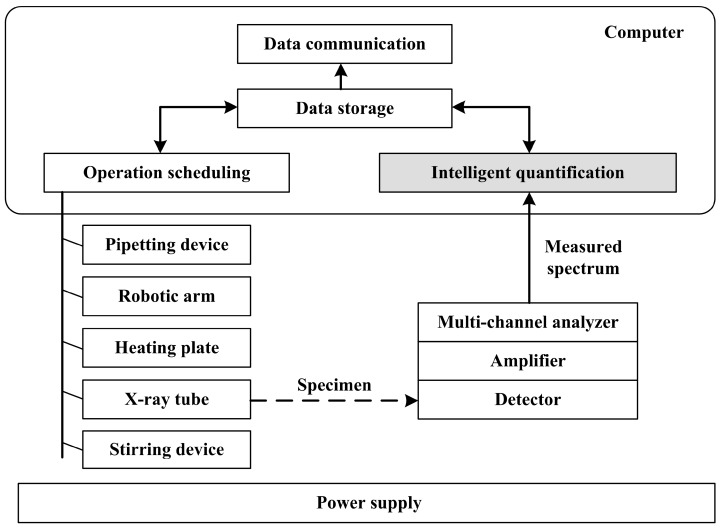
Structure of the online TXRF analysis platform.

The structure of the online TXRF analysis platform is shown in Figure 2. The computer performs operation scheduling, intelligent quantification, data storage, and data communication. The operation scheduling unit controls the components of the platform, including the pipetting device, the robotic arm, the heating plate, the X-ray tube and the stirring device. There are two operation modes, offline mode and online mode. In the offline mode, the test condition is configured, and TXRF calibration can be performed automatically or manually. In the online mode, TXRF measurement is performed periodically. The intelligent quantification unit obtains the measured spectrum and computes the element concentrations, where different quantification methods can be studied. The data storage unit stores calibration and quantification results as well as the settings of test conditions. The data communication unit may send real-time measurement results to a specified monitoring center. Based on this platform, our intelligent quantification method will be discussed.

## 4. Intelligent Quantification Method for Online TXRF Analysis

Focused on the above-mentioned quantification difficulties, an intelligent quantification method is developed for online TXRF analysis. According to the inherent features of measured spectra, a new spectral decomposition framework makes use of characteristic curves, containing all possible peaks of all possible elements from all possible sources and a pre-estimated background curve. The optimization problem for multi-element concentration calculation is formulated. Eventually, the PSO-RBFN-SA algorithm is designed to solve the optimization problem.

### 4.1. Spectral Decomposition Framework

In the basic principle of quantitative multi-element determination, a linear relationship ideally exists between the amount of excited analyte element x, and the intensity of its characteristic peaks, which can be written as:

(2) $$N_{x}=B_{x}C_{x}$$

where $N_{x}$ is the net intensity of the characteristic peaks, $B_{x}$ is a proportionality factor called absolute sensitivity, and $C_{x}$ is the concentration of the analyte element. In TXRF analysis, specimens with a tiny mass and thickness, referring to the sample preparation steps, meet the conditions for this ideal case. However, determination of the net intensity of the characteristic peaks for each analyte element is also difficult.

**Figure 3:**
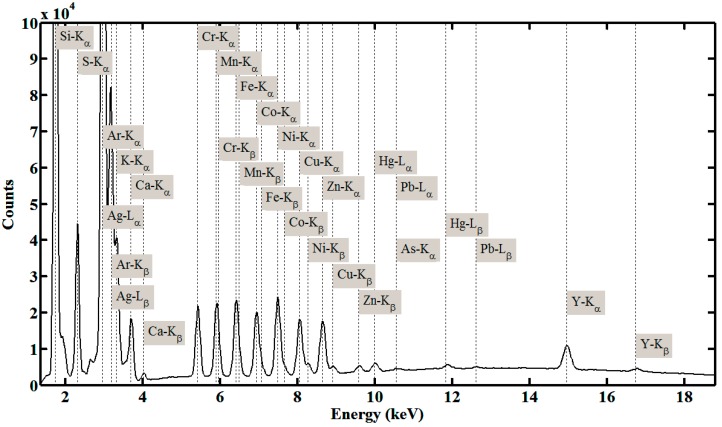
Typical measured spectrum for multi-element determination, where the Ag-anode X-ray tube is operated at 25 kV and 200 μA, the glancing angle is set as 0.09°, and the counting time of detector is set as 600 s.

Figure 3 shows a typical measured spectrum, where a number of elements, such as Si, S, K, Ca, Cr, Mn, Fe, Co, Ni, Cu, Zn, As, Y, Ag, Hg, and Pb, are recognized. Checking the test condition and atmosphere, we can tell that characteristic peaks of Ag are from the target material of the X-ray tube and characteristic peaks of Si are from the quartz sample carrier. Serious overlapping can be found, such as Mn-K_α_ against Cr-K_β_, Fe-K_α_ against Mn-K_β_, Co-K_α_ against Fe-K_β_, Ni-K_α_ against Co-K_β_, Cu-K_α_ against Ni-K_α_, Zn-K_α_ against Cu-K_β_, As-K_α_ against Pb-L_α_, *etc.* When a specimen contains a number of known and unknown elements, overlapping will be a common phenomenon, and also the real spectral background is hard to determine.

The first thing we can do is to organize characteristic peaks from different sources in measured spectra. There are characteristic peaks from analyte elements, characteristic peaks from the measurement atmosphere, characteristic peaks from the component material, scatter peaks from the target material of the X-ray tube, and spurious peaks, including escape peaks and sum peaks, from detector artifacts, which are described, respectively, as follows:

(i) A line spectrum, or a so-called characteristic spectrum, is produced when analyte elements are irradiated with X-rays consisting of a number of characteristic peaks. There are three principal series, the K-, L-, and M-series, which arise when the inner vacancy being filled is in the K-, L-, and M-shell. A series contains several peaks, named α-, β-, and γ-peaks, which mainly differ according to the origin of the outer electron. The relationship of the photon energy (*i.e.*, the peak energy) and analyte element was discovered by H.G.J. Moseley, which can be described as:

(3) $$E_{x}=k_{x}{\left(Z-\sigma_{x}\right)}^{2}$$

where $E_{x}$ is the photon energy, Z is atomic number, $\sigma_{x}$ is interpreted as a shielding constant, and $k_{x}$ is a constant value. In general, the relative intensity of a certain peak in its series is rather similar for most elements.

(ii) Since some TXRF analysis does not take place in vacuum chamber, characteristic peaks from the measurement atmosphere can be found in the measured spectrum. Commonly, characteristic peaks of Ar are produced when argon gas in the air is irradiated with X-ray.

(iii) If the components of the TXRF analysis system are on the X-ray path, the component material, such as Si of the sample carrier as well as Ni and Pb of welding spot near the detector, will bring in corresponding characteristic peaks.

(iv) A small part of the primary beam is deflected from its original direction, *i.e.*, X-ray scatter. There are two different processes. One is called Rayleigh scattering, where an X-ray photon collides with a firmly bound inner electron of an atom, leading to a change of direction of the X-ray photon without energy loss; the other is called Compton scattering, where an X-ray photon collides with a loosely bound outer electron or even with a free electron, leading to a change of direction and a loss of energy. In Compton scattering, an X-ray photon with the energy E keeps the portion $E^{\prime }$ when it is deflected by an angle φ, while the electron takes off the residual part of energy, and the fraction $E^{\prime }/E$ can be calculated as:

(4) $$E^{\prime }/E=1/[1+(1-\cos\varphi )E/E_{e}]$$

where $E_{e}$ is the rest energy of an electron. The primary beam contains characteristic radiation of the target material of the X-ray tube and the continuous spectrum, so the measured spectrum contains Compton peaks and characteristic peaks of target material, as well as a continuous spectrum, which is regarded as the spectral background.

(v) Some additional peaks are regarded as spurious peaks from detector artifacts, including escape peaks and sum peaks. The atoms in the detector also emit their own characteristic radiation when struck by the incoming X-ray beam. The majority of this radiation is immediately absorbed within the detector volume and contributes to the overall energy for the original X-ray photon. However, there is a limited probability that the radiation produced by detector element will escape from the detector volume and not contribute to the energy for the original X-ray photon, resulting in escape peaks. All the peaks described above have the opportunity to generate escape peaks. The escape peaks are located at an energy value lower than their parent peak, equivalent to the difference between the energy of the original peaks and the energy of the characteristic peak of detector element. For example, the Si escape peak is 1.74 keV below the original one. On the other hand, sum peaks appear when two events of high-intensity peaks arrive at the pulse processing electronics of the detector so close together in time that they are not recognized as two events but as only one. Thus, the energy of the sum peak is the sum of the two initial energies. This phenomenon is associated with the count rate limitations in the finite pulse processing time. Fortunately, sum peaks will not happen in our case, because a low power X-ray tube is employed and low concentration samples are tested on our TXRF analysis platform.

**Figure 4:**
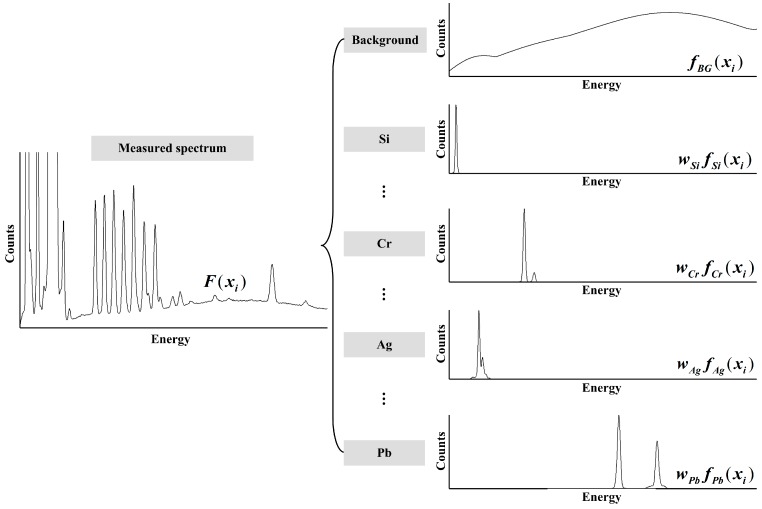
Spectral decomposition framework.

After reviewing the inherent features of measured spectra, a new decomposition framework is developed to determine the net intensity of each analyte element, as shown in Figure 4. In this figure, the measured spectrum is decomposed into one pre-estimated background curve and a number of characteristic curves of possible elements. The background curve can be approximated by exponential polynomial function $f_{BG}(x_{i})$, where the coefficients are to be optimized. The characteristic curve of each element contains corresponding peaks, including characteristic peaks, escape peaks, or Compton peaks. Under the given conditions, the intensity ratio of these peaks is constant for each element (*i.e*., the shape of characteristic curve of each element is constant) and their intensities are proportional to the element concentrations. Therefore, the normalized characteristic curves $f_{element}(x_{i})$ of possible elements can be recorded, and element concentrations can be derived from the proportionality coefficients $w_{element}$ between them and the ones decomposed from the measured spectrum. This spectral decomposition framework is very suitable for multi-element analysis in different scenarios, since the characteristic curve of any element can be easily added into the framework. Quantitative multi-element analysis can be performed with or without qualitative analysis, taking all possible elements into account. Relying on best fitting of measured spectra in the whole spectrum scope, but not just on specific strong peaks, it has potential to obtain high measurement accuracy and robustness.

### 4.2. Formulation of Optimization Problem

According to the spectral decomposition framework, the measured spectrum is regarded as a combination of one background curve and a number of characteristic curves. Assuming that there are m elements in the spectral decomposition framework, it can be written as:

(5) $$F(x_{i})\overset{decompose}{\to }f_{BG}(x_{i})+\sum_{j=1,2,\ldots ,m}w_{j}f_{j}(x_{i})\;i=1,2,\ldots n$$

where $x_{i}$ is the energy value, F is the curve of measured spectrum, $F_{BG}$ is the pre-estimated background curve, $w_{j}$ is the proportionality coefficients for element j, which can be regarded as a weight, and $f_{j}$ is the normalized characteristic curve of element j.

The background curve is approximated by exponential polynomial function, which is written as:

(6) $$f_{BG}(x_{i})=a_{0}\exp\left[\sum_{\lambda =1,2,\ldots ,\tau }a_{t}{\left(x_{i}-\bar{x}\right)}^{\lambda }\right]\;i=1,2,\ldots n$$

where $a_{0}$ and $a_{t}$ are exponential polynomial coefficients, τ is the highest power which is set as a constant, $\bar{x}$ is the medium energy value in the whole spectrum scope.

Assuming that the characteristic curve of element j contains n peaks, including characteristic peaks, escape peaks, or Compton peaks, each peak is approximated by a Gaussian function. Then, the normalized characteristic curve of element j can be defined as:

(7) $$f_{j}(x_{i})=\sum_{k=1,2,\ldots ,n}g_{j}^{k}(x_{i})/\underset{k=1,2,\ldots ,n}{\text{MAX}}(A_{j}^{k})=\sum_{k=1,2,\ldots ,n}A_{j}^{k}\exp\left[-\frac{\left(x_{i}-E_{j}^{k}\right)}{2\sigma_{j}^{k}}\right]/\underset{k=1,2,\ldots ,n}{\text{MAX}}(A_{j}^{k})$$

where $g_{j}^{k}$ is the Gaussian function representing the k-th peak of element j, $A_{j}^{k}$ is the height of the k-th peak of element j, $\sigma_{j}^{k}$ is the width of the k-th peak of element j, $E_{j}^{k}$ is the center energy value of the k-th peak of element j. $\sigma_{j}^{k}$ can be derived from the experimental FWHM value, $A_{j}^{k}$ can be calculated by adopting the known intensity ratio of peaks. In this way, the normalized characteristic curve of each element contains possible peaks, so it can be considered as a sophisticated model for the net intensity.

Hence, the problem becomes finding the optimal combination of exponential polynomial coefficients and proportionality coefficients to fit the measured spectrum. Here the least squares criterion is utilized to evaluate the fitting residual. The objective function of combinatorial optimization problem can be formulated as:

(8) $$z(a_{0},a_{1},\ldots ,a_{\tau },w_{1},w_{2},\ldots ,w_{m})=\sum_{i=1,2,\ldots ,n}{\left[F(x_{i})-f_{BG}(x_{i})-\sum_{j=1,2,\ldots ,m}w_{j}f_{j}(x_{i})\right]}^{2}$$

Then the optimization goal is to minimize the multivariate function z, looking for the optimal solution of $\left[a_{0},a_{1},\ldots ,a_{\tau },w_{1},w_{2},\ldots ,w_{m}\right]$. In this manner, the background curve is optimized, avoiding over-fitting or under-fitting. Besides, constraints of the background curve shall be considered to lower the order of solution space. For example, it is assumed that the data point (x,F(x)) on the flat part of the measured spectrum is also on the background curve, which can be written as:

(9) $$F(x)=f_{BG}(x)=a_{0}\exp\left[\sum_{t=1,2,\ldots ,\tau }a_{t}{\left(x-\bar{x}\right)}^{t}\right]$$

Since the measured spectrum F cannot be described by a certain function, a numerical computation method should be considered to perform optimization. Intelligent computing methods [56,57,58] can be applied to solve the combinatorial optimization problem, which will be discussed in the next section.

With the optimization results during calibration and measurement, multi-element concentrations can be calculated. According to Equation (2), a calibration straight line describing the relationship between the measured net intensity and the concentration of each element should be given before calculation of multi-element concentration. Since different elements generally have different sensitivities, relative sensitivities with reference to a specific element (*i.e*., a reference element) can be determined by calibration. The relative sensitivities have to be measured only after the installation of the TXRF analysis system or after a modification. During the calibration, the relative sensitivity of element j can be calculated as:

(10) $$S_{j}=\frac{N_{j}^{c}/C_{j}^{c}}{N_{rf}^{c}/C_{rf}^{c}}\times S_{rf}$$

where $N_{j}^{c}$ is the net intensity of element j during calibration, $N_{rf}^{c}$ is the net intensity of reference element during calibration, $C_{j}^{c}$ is the concentration of element j during calibration, $C_{rf}^{c}$ is the concentration of reference element during calibration, and $S_{rf}$ is the relative sensitivity of reference element which can be set to 1.

Similar to the traditional approaches, internal standardization can be applied to determine the multi-element concentration. The concentration of element j can be calculated as:

(11) $$C_{j}=\frac{N_{j}/S_{j}}{N_{is}/S_{is}}\times C_{is}$$

where $N_{j}$ is the net intensity of element j during measurement, $N_{is}$ is the net intensity of the internal standard during measurement, $S_{is}$ is the relative sensitivity of the internal standard, and $C_{is}$ is the concentration of the internal standard during measurement.

Fortunately, any stable element that does not exist in the specimen can be chosen flexibly as the reference element or the internal standard in our method. The reference element and the internal standard can be the same element and related concentration is constant during calibration or measurement. The spectral decomposition is performed during both calibration and measurement, and the proportionality coefficients indicate the net intensities. Hence, the concentration of element j can be calculated as:

(12) $$C_{j}=\frac{N_{j}N_{is}^{c}}{N_{j}^{c}N_{is}}\times C_{j}^{c}=\frac{w_{j}w_{is}^{c}}{w_{j}^{c}w_{is}}\times C_{j}^{c}$$

where $N_{is}^{c}$ is the net intensity of the internal standard during calibration, $w_{j}$ is the proportionality coefficient of element j during measurement, $w_{j}^{c}$ is the proportionality coefficient of element j during calibration, $w_{is}$ is the proportionality coefficient of the internal standard during measurement, and $w_{is}^{c}$ is the proportionality coefficient of the internal standard during calibration. In Equation (12), $C_{j}^{c}$, $w_{j}^{c}$ and $w_{is}^{c}$ can be regarded as constants, so it can be written as:

(13) $$\frac{w_{j}}{w_{is}}=\frac{w_{j}^{c}}{w_{is}^{c}C_{j}^{c}}\times C_{j}$$

which is similar to Equation (2). Here, $w_{j}/w_{is}$, which we call the relative net intensity of element j, depends linearly on the element concentration. In this way, a calibration is needed once to determine $w_{j}^{c}$ and $w_{is}^{c}$, utilizing multi-element standards or mixed single-element standards, and then we only need to obtain $w_{j}$ and $w_{is}$ in each measurement to calculate the element concentration. Since the procedure of spectral decomposition is exactly the same during calibration and measurement, the measurement accuracy is guaranteed.

### 4.3. PSO-RBFN-SA Algorithm

Considering the requirement of online TXRF analysis, a precise, robust and rapid algorithm should be designed to solve the optimization problem described in Equation (8). Some optimization algorithms, such as genetic algorithms (GA), are discussed in combinatorial optimization problems. However, in this case, where the solution dimension is extremely high and search granularities on different dimensions differ, the global optimization ability of such algorithms is limited. Kennedy *et al.* developed particle swarm optimization (PSO) in 1995 based on the analogy of swarms of birds and schools of fish [4,5]. PSO is an efficient optimization tool for solving combinatorial optimization and dynamic optimization problems. Like other evolutionary algorithms, PSO uses fitness as criterion to evolve the behavior of the solution population. Potential solutions, namely particles, fly through the search space. Each particle keeps track of the best position it has achieved so far, which represents a particle experiment. Another kind of experiment is the best position that has been achieved by any companion of the particle so far. The particle velocity is constantly adjusted according to the two kinds of experiments.

Since global optimization is time consuming and short computing time is a critical demand of online analysis, we propose a PSO-RBFN-SA algorithm. It has two phases, an offline phase and an online phase. During the offline phase, global optimization is performed on spectra of multi-elements of different concentrations, measured or simulated, and then Radial basis function network (RBFN) is utilized to store the optimization results. RBFN presents good approximation properties [6]. The RBFN family is broad enough to uniformly approximate any continuous function on a compact set. Radial basis functions are a special class of function, of which responses decrease (or increase) monotonically with the distance from a central point. In principle, they could be employed in any sort of linear or nonlinear model and single-layer or multi-layer network. RBFN is a three-layer feed-forward neural network, which is embedded with several radial-basis functions. Such a network is characterized by an input layer, a single layer of non-linear processing neurons, and an output layer. RBFN can approximate the continuous function between spectra and global optimization results. During the online phase, TXRF measurement runs periodically, and the global optimization results of each online measured spectrum can immediately be inferred by RBFN. In addition, simulated annealing (SA) is used to enhance the optimization accuracy, which has a strong ability of finding the local optimal result [7]. SA mainly consists of the repeating of two steps: a generation mechanism and an acceptance criterion. It starts off at an initial state with a high temperature, and then a sequence of iterations is generated. A perturbation mechanism transforms the current state into a next state selected from the neighborhood of the current state. If this neighboring state has better fitness, the neighboring state is accepted as the current state. If this neighboring state has worse fitness, the neighboring state is accepted with a certain probability determined by the acceptance criterion. After sufficient times of acceptance, the temperature is decreased. This process is repeated until the final temperature is reached. In this way, the measurement accuracy is ensured and computing time is saved for online analysis. The pseudo-code for PSO-RBFN-SA is outlined as follow.

**Algorithm 1.** PSO-RBFN-SAOffline phase$n_{spectrum}$ prepared spectra are used as samples for RBFN training.For
$\alpha =1,2,\ldots ,n_{spectrum}$ PSO is performed to the spectrum
$F_{\left(\alpha \right)}$, and the population of particles is set as
$n_{particle}$. For
$l=1,2,\ldots ,n_{particle}$  $P_{l}$
represents the current solution, which is initialized as a random solution in the solution space:
(14)$$P_{l}(1)=[a_{0},a_{1},\ldots ,a_{\tau },w_{1},w_{2},\ldots w_{m}]$$  $V_{l}$
represents the current velocity, which is initialized as a random velocity:
(15)$$V_{l}(1)=[v_{1},v_{2},\ldots ,v_{\tau +m}]$$  $P_{l}^{s}$
represents the best solution it has achieved so far, which is initialized as:
(16)$P_{l}^{s}(1)=P_{l}(1)$ End The maximum iteration of PSO is set as
$n_{PSO}$. For
$t=1,2,\ldots ,n_{PSO}$  The global best solution
$P^{g}$
is defined as:
(17)$$z(P^{g}(t))=\text{MIN}(z(P_{l}^{s}(t)))\;l=1,2,\ldots ,n_{particle}$$  For
$l=1,2,\ldots ,n_{particle}$   The weighted particle velocity is updated as:
(18)$$V_{l}(t+1)=\eta (t)V_{l}(t)+c_{1}R_{1}[P_{l}^{s}(t)-P_{l}(t)]+c_{2}R_{2}[P^{g}(t)-P_{l}(t)]$$
where
$R_{1}$
and
$R_{2}$
are two separate random number between 0 and 1, while
$c_{1}$
and
$c_{2}$
are acceleration constants, representing the weight of acceleration terms that pull each particle toward the local best solution and the global best solution.
η(t)
is the inertia weight for balancing the global and local influences, which is defined as:
(19)$$\eta (t)=0.9-\frac{t}{n_{PSO}}\times 0.5$$
where
η(t)
linearly decreases through the course of the run. A large inertia weight facilitates a global search while a small inertia weight facilitates a local search. Accordingly, the optimization process can converge to the neighborhood of global optimal solution smoothly at the prophase and converge to the global optimal solution quickly at the anaphase.   The solution of each particle is updated as:
(20)$P_{l}(t+1)=P_{l}(t)+V_{l}(t+1)$   The best position of particle is calculated as:
(21)$$P_{l}^{s}(t+1)=\{\begin{array}{cc} P_{l}^{s}(t) & z(P_{l}(t+1))\geq z(P_{l}^{s}(t))\\ P_{l}(t+1) & z(P_{l}(t+1))<z(P_{l}^{s}(t)) \end{array}$$  End End The global optimization result of
$F_{\left(\alpha \right)}$
is recorded as
$P_{\left(\alpha \right)}^{g}$.End RBFN is trained with the optimization results, where the training inputs are defined as:
(22)$$X_{\left(\alpha \right)}=[F_{\left(\alpha \right)}(x_{1}),F_{\left(\alpha \right)}(x_{2}),\ldots ,F_{\left(\alpha \right)}(x_{n})]\;\alpha =1,2,\ldots ,n_{spectrum}$$
and the training outputs are defined as:
(23)$Y_{\left(\alpha \right)}=P_{\left(\alpha \right)}^{g}\;\alpha =1,2,\ldots ,n_{spectrum}$Online phase The global optimization result of each online measured spectrum
F
is inferred by the constructed RBFN, where the input is set as:
(24)$X=[F(x_{1}),F(x_{2}),\ldots ,F(x_{n})]$ The output is calculated as:
(25)$$Y=\sum_{\beta =1，2，\ldots ,N}\omega_{\beta }\psi (\left\|X-c_{\beta }\right\|)$$
where
ψ
is a basis function,
‖•‖
denotes the Euclidean norm,
$\omega_{\beta }$
is the weight in the output layer,
N
is the number of neurons in the hidden layer, and
$c_{\beta }$
is the center of RBF in the input vector space. SA is performed with the initial solution
Y, and the initial state
A
is defined as
Y. Initial temperature is set as
T, and the maximum iteration of SA is set as
$n_{SA}$.For
$t=1,2,\ldots ,n_{SA}$ The cooling condition is that the best state remains unchanged for
K times. While the cooling condition is not satisfied  Use a perturbation mechanism to generate a new state
$A^{\prime }$:
(26)$$A^{\prime }=A+randn•\Delta$$where
randn
is a normally distributed random number.
Δ
is defined as:
(27)$$\Delta =[\delta_{1},\delta_{2},\ldots ,\delta_{\tau +m}]$$
and $\delta_{\gamma }$ (γ=1,2,…,τ+m) is defined with a random integer
$\gamma_{0}$
in
[1,τ+m]:
(28)$$\delta_{\gamma }=\{\begin{array}{cc} 1 & \gamma =\gamma_{0}\\ 0 & \gamma \neq \gamma_{0} \end{array}$$  The decrease of fitness is:
(29)$dz=z(A^{\prime })-z(A)$  Check whether the new state should be accepted according to Metropolis criteria:
(30)$$A=\{\begin{array}{cc} A^{\prime } & df<0\text{ or }\exp(-\frac{df}{\kappa T})>rand\\ A & \text{else} \end{array}$$
where
κ
is Boltzmann constant and
rand
is a random number in
[0,1]. End Cool down with a parameter
ρ:
(31)T=ρTEndThe result of
$\left[a_{0},a_{1},\ldots ,a_{\tau },w_{1},w_{2},\ldots w_{m}\right]$
is returned to calculate multi-element concentrations.

## 5. Experimental Results

In this section, we run online TXRF analysis and evaluate the performance of the intelligent quantification method.

### 5.1. Experimental Settings

Measurement is carried out on the established online TXRF analysis platform. The measurement period is set as 30 min. The Ag-anode X-ray tube is operated at 25 kV and 200 μA, while the glancing angle is set as 0.09°. For the detector, counting time is set as 600 s. The energy range for data processing is from 1.354 keV to 17.920 keV. We mainly focus on the quantitative determination of trace heavy metals, including Cr, Mn, Fe, Co, Ni, Cu and Zn, while still 18 elements, including Si, S, Cl, Ca, Cr, Mn, Fe, Co, Ni, Cu, Zn, As, Rb, Zr, Ag, Hg, Pb and Bi, are considered in the spectral decomposition framework. In approximating the background curve, the highest power of exponential polynomial function is set as 6.

Single-element standards and multi-element standards are used to prepare aqueous solutions as samples. All chemicals used in this study are analytical grade reagents. Thirty microliters of sample solution is taken to prepare each single specimen.

In the PSO-RBFN-SA algorithm, 600 simulated spectra are used as samples for RBFN training, the particle number $n_{particle}$ is set as 50, the PSO iteration number $n_{PSO}$ is set as 200, the acceleration constants $c_{1}=c_{2}=1$, the SA iteration number $n_{SA}$ is set as 40, parameter K is set as 4, Boltzmann constant κ is 1, and parameter ρ is set as 0.6.

### 5.2. Performance Evaluation

**Figure 5:**
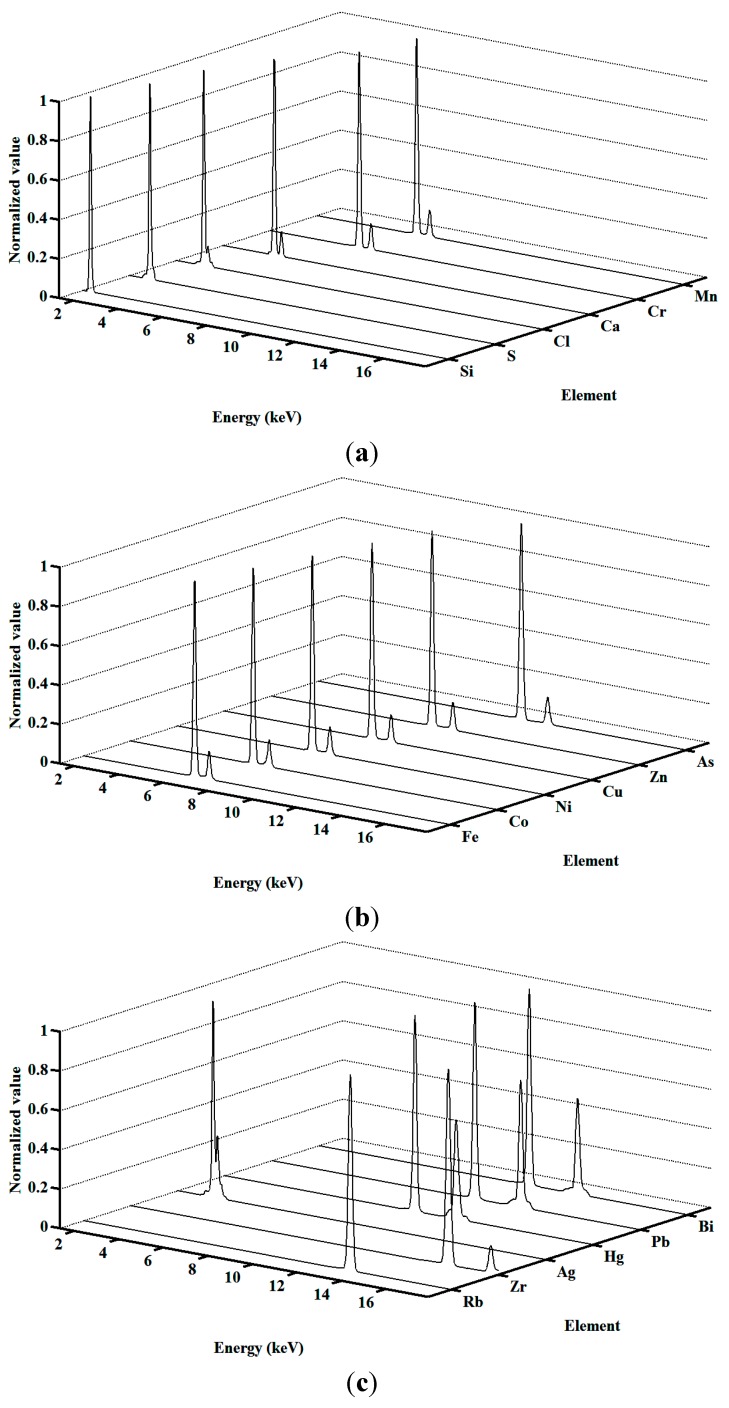
Normalized characteristic curves in the spectral decomposition framework. (**a**) Normalized characteristic curves of Si, S, Cl, Ca, Cr and Mn; (**b**) Normalized characteristic curves of Fe, Co, Ni, Cu, Zn and As; (**c**) Normalized characteristic curves of Rb, Zr, Ag, Hg, Pb and Bi.

As shown in Figure 5, the normalized characteristic curves of Si, S, Cl, Ca, Cr, Mn, Fe, Co, Ni, Cu, Zn, As, Rb, Zr, Ag, Hg, Pb and Bi are obtained. Details of characteristic peaks, escape peaks or Compton peaks are recorded in these characteristic curves. In this figure, the normalized characteristic curves of heavy metals, Cr, Mn, Fe, Co, Ni, Cu and Zn, are for quantitative analysis. The normalized characteristic curves of Si and Ag are used to approximate the intensities coming from the quartz sample carrier and target material of the X-ray tube, respectively. The normalized characteristic curves of S, Cl, Ca, As, Rb, Zr, Hg, Pb and Bi are acquired to guarantee the fitting accuracy, and also to demonstrate the robustness of the proposed method.

**Figure 6:**
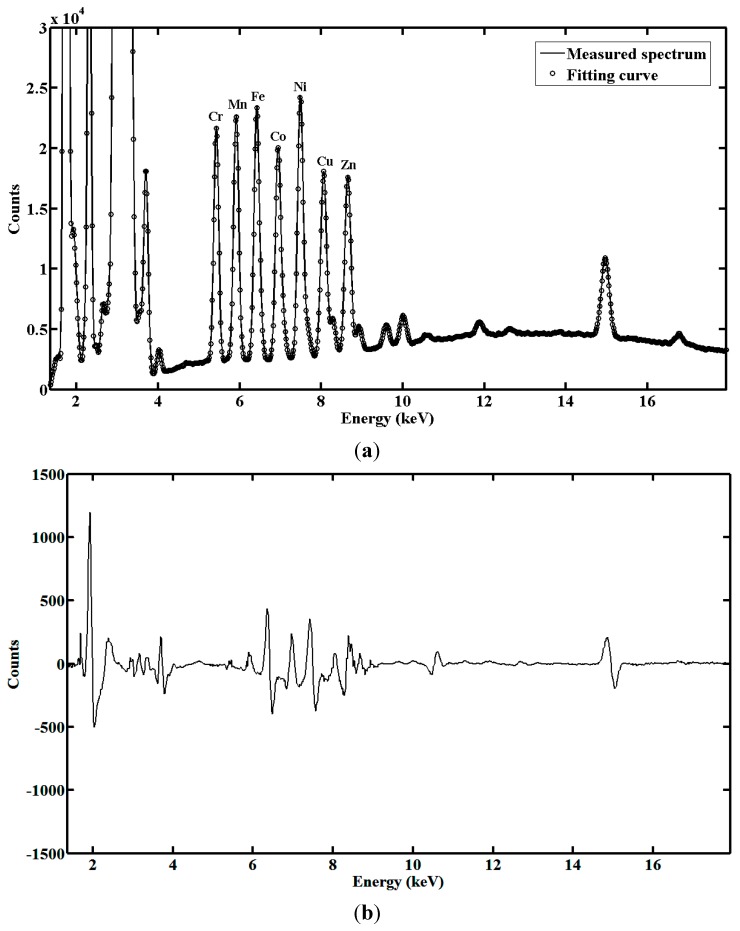
Fitting curve of measured spectrum obtained by PSO-RBFN-SA. (**a**) Comparison between measured spectrum and its fitting curve; (**b**) Fitting residual curve.

Based on the spectral decomposition framework, the best fitting curve of each online measured spectrum is found by PSO-RBFN-SA. Testing a water sample with 8 mg/L of Cr, Mn, Fe, Co, Ni, Cu and Zn, one measured spectrum and corresponding fitting curving is shown in Figure 6, and the fitting residual curve is given as well. It can be seen that the fitting curve matches the measured spectrum well. The absolute value of fitting residual at energy from 5 keV to 11 keV, which is related to the measurement accuracy of heavy metals of interest, is lower than 500 counts. This value is lower than the 800 counts in tested samples. Thus, high precise approximation of measured spectra is achieved by the proposed method.

**Figure 7:**
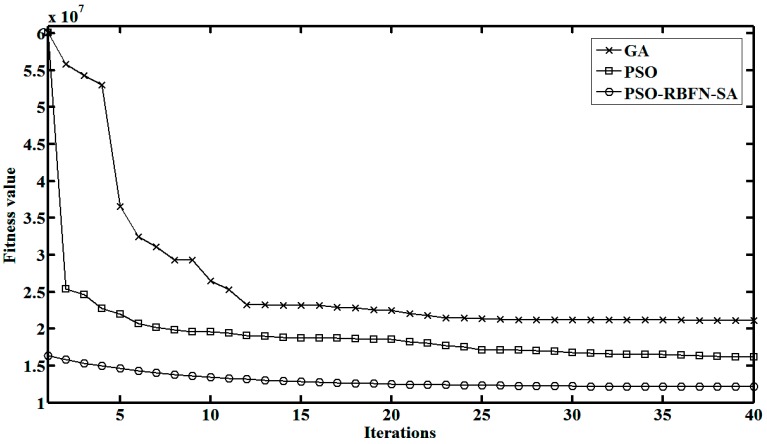
Convergence curves of GA, PSO and PSO-RBFN-SA during online phase.

Optimization capability of GA, PSO and PSO-RBFN-SA is compared in Figure 7. These algorithms are employed in the online TXRF analysis, testing the water sample referred in Figure 6. It shows that the convergence of PSO is faster than that of GA. Since the global optimization is done in the offline phase, and the local optimization is aided by SA, PSO-RBFN-SA has the best initial solution and the best final results during the online phase. It is demonstrated that the PSO-RBFN-SA algorithm promotes the optimization efficiency.

**Figure 8:**
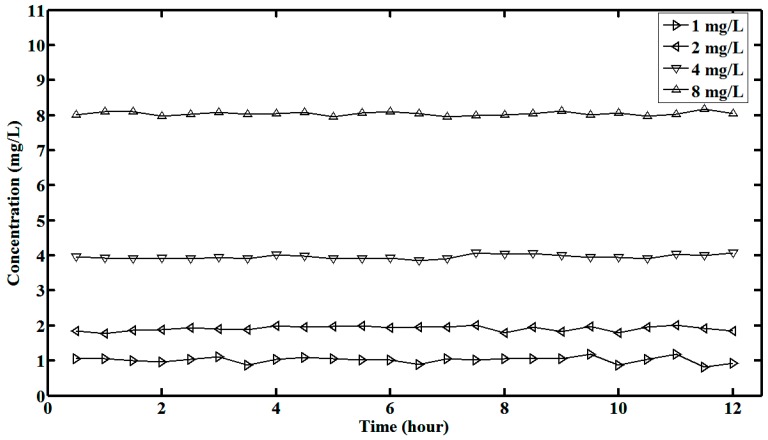
Online measurement results of Cr for 12 h.

Utilizing water samples with different concentrations (1 mg/L, 2 mg/L, 4 mg/L and 8 mg/L) of Cr, Mn, Fe, Co, Ni, Cu and Zn, simultaneous quantitative online analysis of these heavy metals of interest is performed for 12 h, while the measurement period is set as 30 min. Twenty-four sets of measurement results are obtained for each water sample. The online measurement results of Cr are shown in Figure 8. We can find that the performance of TXRF analysis is not degraded over time.

**Figure 9:**
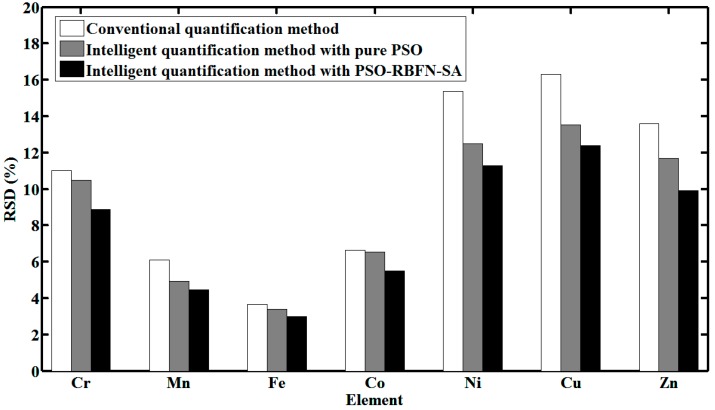
Comparison of conventional and proposed quantification methods in accuracy of measurement results.

Moreover, experiments are carried out to evaluate the measurement accuracy of the proposed intelligent quantification method. Three cases are compared here. The first is to measure heavy metals of interest with PSO-RBFN-SA. The second is to measure heavy metals of interest with pure PSO. The third is to measure heavy metals of interest with the conventional method manually, where the net intensities of elements are acquired from separated strong characteristic peaks. For water sample with 1 mg/L of Cr, Mn, Fe, Co, Ni, Cu and Zn, Figure 9 shows the relative standard deviation (RSD) of measurement results in three cases.

It is verified that PSO-RBFN-SA contributes to more accuracy in measurement results. It can be concluded that the proposed intelligent quantification method can realize robust online TXRF analysis, and it can also achieve higher measurement accuracy than the conventional method.

## 6. Conclusions

To satisfy the urgent requirement of simultaneous quantitative online analysis of trace heavy metals from dynamic environmental monitoring and management, we establish a brand new online TXRF analysis platform. Focused on problems of spectral overlapping and background effects, as well as critical demands of online analysis, this paper proposes an intelligent quantification method for online TXRF analysis. A scalable spectral decomposition framework is developed where the measured spectrum is regarded as a combination of one background curve and a number of characteristic curves of all possible elements in the whole spectrum scope. Then, the quantification becomes an optimization problem of curve fitting. A hybrid algorithm, PSO-RBFN-SA, is well designed for online TXRF analysis in order to ensure measurement accuracy and save computing time, with offline global optimization and online fast computing. From the experiments, the onine TXRF analysis scheme is demonstrated to be efficient, and the proposed intelligent quantification method achieves high measurement accuracy with short online computing time. The main contribution of this paper is that TXRF analysis is extended to the online monitoring domain; the scalable spectral decomposition framework facilitates simultaneous quantitative online analysis of multi-element; and PSO-RBFN-SA is designed specifically for online TXRF analysis. In future research, objective function shall be evolved further, considering different fitting accuracies in different spectrum areas, and more flexible models of net intensities shall be investigated, instead of shape-fixed characteristic curves. In addition, this intelligent quantification method for online TXRF analysis will be tested in scenarios of other samples and applications.
